# Supporting people with dementia to live at home

**DOI:** 10.1186/s12877-023-04389-w

**Published:** 2023-10-19

**Authors:** Monica Leverton, Patrick Pui Kin Kor

**Affiliations:** 1 NIHR Policy Unit in Health and Social Care Workforce, King’s College London, London, UK; 2 School of Nursing, The Hong Kong Polytechnic University, Hong Kong, China

## Main text

Dementia prevalence is predicted to rise from 57.4 million cases globally in 2019, to 152.8 million cases by 2050 [1]. The considerable increase in the number of people living with dementia reflects the need for care and support planning efforts to improve the lives of people affected by dementia.

Many people with dementia prefer to continue living in their own homes, despite advancing symptoms and cognitive decline. This is in contrast to finding alternative living arrangements where health and social care can be more readily available, such as extra care housing, or a residential care facility. A move to these settings can be experienced as a loss of independence for people living with dementia, and associated with feelings of guilt by family carers, particularly when this decision is made on behalf of the individual [2]. Consequently, moving out of home can be seen as a last resort for people affected by dementia, when other sources of support no longer work.

For many people affected by dementia, the home represents a site of familiarity, comfort, and safety [3]. However, living at home is often associated with a higher level of risk, including risks to safety (i.e. increased risk of falls), physical health (i.e. self-neglect and medication mismanagement), and psychological wellbeing (i.e. related to social isolation and loneliness). In this collection, we explore current research around best practice and solution-focussed approaches to enable people with dementia to live at home and continue to age in place, with the right support.

At an individual-level, people living with dementia may be supported to remain independent at home with help from telecare, assistive technology (including gerontechnology), home modifications, and home-based interventions. Such interventions have the potential to aid memory, support activities of daily living (ADLs), facilitate self-management, and lower risk of falls in the home [4]. However, individual-level support sources typically focus on risk prevention and safety of individuals living with dementia, giving less consideration to maintaining or improving quality of life and wellbeing. There is also uncertainty around the effectiveness and suitability of these interventions for individuals with more advanced stages of dementia. More evidence is needed to understand the scope, and overall effectiveness of individual-level sources of support to help people with dementia to live at home for longer, and with good quality of life. We hope to see articles expand on these themes in this collection.

At a systemic-level, people with dementia can be supported to live at home by informal and/or formal caregivers. Informal (unpaid) care equates to half the global cost of dementia, with higher rates of informal caregiving in low and middle income countries [5]. Providing dementia care can be experienced as overwhelming, particularly without the right support in place for both the individual, and the caregiver. Informal caregivers report challenges including finding ways to balance their caregiving tasks with other demands, such as their career, family, and social life. The impact of caregiving on informal caregivers is widely reported, with negative implications of financial, social and psychological stressors, as well as negative associations for care recipients, including premature institutionalization [6].

Finding ways to improve caregiver well-being may help people with dementia to live at home for longer, delaying or preventing a move to long-term residential care. A range of psychosocial interventions have been designed to support informal caregivers, including respite care, support group, mindfulness training, and caring skills training to alleviate caregiving stress and burden [7]. However, questions exist around the sustainability of the benefits beyond the life of the programs. For example, whilst caregivers may take temporary relief when using respite services, the effect on stress may only be transient [8]. To prevent a decline in the physical and mental health, and social well-being of caregivers, an assessment of their needs is required, with provisions for multisectoral care, support and services in place.

People with dementia can also be supported to live at home with help from formal (paid) care workers. As frontline workers, such support can include providing direct care to people in their own homes, also known as domiciliary care. Whilst this can be a vital source of support for people living with dementia and their informal caregivers, formal carers face challenges of their own. Homecare workers often receive limited training to support the needs and wellbeing of people living with dementia, and have unsatisfactory working conditions such as low pay, minimal support, and erratic working schedules. Formal caregivers are often portrayed as low-skilled workers, leading to difficulties in recruiting adequate numbers of care staff [9]. Interventions to support homecare workers focus on training to improve relational and emotional skills, communication, and understanding of client behaviours, as well as increased support with navigating the homecare role and identity, including drawing on organisational and peer support [10]. To mitigate these challenges, further studies should focus on ways to improve homecare workers’ dementia knowledge and skills, in addition to providing them with greater systemic support, good working conditions, and recognition.

A key message of this collection is therefore the importance of supporting those who provide care to older people living with dementia, enabling them to live at home for as long as possible. We encourage articles exploring solution-focussed approaches for both informal and formal caregivers.

At a broader level, community-based approaches are steering towards the ongoing development of dementia-friendly neighbourhood and community initiatives. To date, these have included public education programmes and awareness raising, inclusive environmental design, and active involvement of people affected by dementia in their communities and groups. Adopting a rights-based approach, these initiatives aim to promote social inclusion for people affected by dementia, addressing their needs through improved social and physical environments to enable their participation in the community for as long as possible. The role of caregivers is also integral to these approaches. Such initiatives should be explored further to expand the evidence-base in understanding the scope and possibilities of community-based approaches to support people affected by dementia.

It is, however, important to conclude in stating that living at home is not always the best or most appropriate option for people affected by dementia. Individuals and their caregivers need support identifying when home is no longer best, with help towards advanced care planning and in making alternative arrangements that feel right for them. Comprehensive assessments for both the person living with dementia and their caregivers can provide important information to facilitate decision-making and advanced planning. Several needs assessment tools have been developed for dementia care, however the majority of these focus on the practical needs of the individual and their caregivers (e.g. finance, physical health), instead of variables such as wellbeing (e.g. loneliness), or the suitability of the home environment itself.

It is important for people living with dementia to be able to age in place, in a way that they, and their caregivers choose. Finding effective means of supporting older people with dementia and their caregivers to live well is a priority for dementia research. The aim of this collection is therefore to identify effective approaches, mechanisms, and systems to support older people with dementia to continue to live in their own homes, for as long as is wanted, and warranted. We believe this collection is highly relevant for anyone involved in the planning of dementia care and support.

## Data Availability

Not applicable.

## Abbreviations

ADLs: Activities of Daily Living
